# Associations between Changes in Food Acquisition Behaviors, Dietary Intake, and Bodyweight during the COVID-19 Pandemic among Low-Income Parents in California

**DOI:** 10.3390/nu15214618

**Published:** 2023-10-31

**Authors:** Gail Woodward-Lopez, Erin Esaryk, Suzanne Rauzon, Sridharshi C. Hewawitharana, Hannah R. Thompson, Ingrid Cordon, Lauren Whetstone

**Affiliations:** 1 Nutrition Policy Institute, Division of Agriculture and Natural Resources, University of California, 1111 Franklin Street, Eleventh Floor, Oakland, CA 94607, USA; eesaryk@berkeley.edu (E.E.); srauzon@ucanr.edu (S.R.); shewawitharana@ucanr.edu (S.C.H.); 2 Department of Epidemiology and Biostatistics, University of California San Francisco, 550 16th Street, 2nd Floor, San Francisco, CA 94158-2549, USA; 3 School of Public Health, University of California, Berkeley, 2121 Berkeley Way West, Suite 6120, Berkeley, CA 94720, USA; thompsonh@berkeley.edu; 4 Research, Evaluation, and Strategic Alignment Section of the Nutrition Education and Obesity Prevention Branch (NEOPB), Center for Healthy Communities, California Department of Public Health, 1616 Capitol Avenue, Sacramento, CA 95814, USA; ingrid.cordon@cdph.ca.gov (I.C.); lauren.whetstone@cdph.ca.gov (L.W.)

**Keywords:** nutrition, Supplemental Nutrition Assistance Program, California, COVID-19, food behaviors, adults

## Abstract

COVID-19 disrupted food access, potentially increasing nutritional risk and health inequities. This study aimed to describe and assess associations between changes in food/meal acquisition behaviors and relative changes in dietary intake and bodyweight from before to during the pandemic. Low-income parents (n = 1090) reported these changes by online survey in April–August 2021. Associations were assessed by multinomial logistic regression. Compared to those with no change, those who decreased supermarket shopping had greater odds of decreased fruit and vegetable (FV; OR[95%CI] = 2.4[1.4–4.1]) and increased salty snack intakes (OR[95%CI] = 1.7[1.0–2.8]). Those who decreased farmer’s market shopping had greater odds of decreased FV intake (OR[95%CI] = 1.8[1.0–3.1]), increased bodyweight (OR[95%CI] = 1.7[1.1–2.6]), and increased SSB (OR[95%CI] = 1.9[1.1–3.2]) and sweets intakes (OR[95%CI] = 1.8[1.1–2.9]). Those who increased online food ordering had greater odds of increased sweets (OR[95%CI] = 1.7[1.1–2.8]), salty snacks (OR[95%CI] = 1.9[1.2–3.2]), and fast food (OR[95%CI] = 2.0[1.2–3.5]) intakes and bodyweight (OR[95%CI] = 1.8[1.1–2.9]). Those who increased healthy meal preparation had greater odds of increased FV intake (OR[95%CI] = 4.0[2.5–6.5]), decreased SSB (OR[95%CI] = 3.7[2.3–6.0]), sweets (OR[95%CI] = 2.7[1.6–4.4]), salty snacks (OR[95%CI] = 3.0[1.8–5]) and fast food intakes (OR[95%CI] = 2.8[1.7–4.6]) and bodyweight (OR[95%CI] = 2.2[1.2–4.0]). Interventions to address the potentially negative impacts of online food/meal shopping and support healthy home cooking may be needed to improve nutrition-related outcomes and reduce health disparities in the aftermath of the current pandemic and during future emergencies requiring similar restrictions.

## 1. Introduction

The importance of a healthy diet for the prevention of chronic diseases, such as cardiovascular disease, cancers, diabetes, and obesity, is well established [1,2]. Evidence suggests that some foods, including sugar-sweetened beverages (SSBs), fast foods, and high-sugar and high-fat snack foods, can lead to calorie intake in excess of energy needs, leading to weight gain and obesity [2]. Obesity and poor dietary intake are widespread in the U.S. and disproportionately affect low-income individuals and communities of color [3].

SARS-CoV-2 (COVID-19) was identified in the United States in January 2020. As of May 2022, there were over 84 million COVID-19 cases and approximately 1 million deaths [4], with low-income and communities of color experiencing disproportionately negative impacts [5]. Poor nutrition and weight status have been shown to be associated with the risk of contagion and complications from COVID-19 [6,7]. Therefore, it is important to understand changes in nutrition-related behaviors, such as food acquisition and consumption behaviors, during the pandemic in order to inform programs and policies to support healthy eating and reduce health disparities.

In California, COVID-19-related shelter-in-place orders were issued statewide beginning in March 2020 and continued in much of the state through June 2021. Schools, childcare centers, and worksites were closed, and activities were restricted to those deemed essential, such as grocery shopping or caring for relatives [6,8]. Restrictions were lifted and reinstated as new virus variants caused waves of infection through 2021 into 2022. These restrictions led to economic and social disruptions, which affected food access due to retail outlet closures and supply chain issues, thereby increasing nutritional risk [9,10]. Studies found that during COVID-19 lockdowns, body mass index (BMI) increased and was associated with increased snacking, consumption of ultra-processed foods, and emotional eating [11,12,13]. Additional research demonstrated that pandemic disruptions led to decreased spending on food-away-from-home [14], reduced in-person shopping and increased online shopping [15], and increased food prepared and consumed at home [16,17]. However, none of these studies examined how these changes in food shopping behavior affected dietary intake during the pandemic, nor did they focus on low-income populations that face greater risk for poor nutrition, food insecurity, obesity [3], and COVID-19 infection and complications [5].

Given these COVID-19-related changes in food access and increases in nutritional risk that could disproportionately affect low-income populations and communities of color, information on changes in food acquisition and dietary intake during the pandemic is needed to inform nutrition programs, such as the Supplemental Nutrition Assistance Program Education (SNAP-Ed). SNAP-Ed is the education component of SNAP (a federal program that provides funding for food purchases for income-qualifying households). SNAP-Ed is the largest on-going nutrition education and obesity prevention program in the U.S., reaching millions of low-income Californians [18] and millions more nationwide. A better understanding of the changes in food-related behaviors among SNAP-Ed-eligible populations (those with incomes at or below 185% of the Federal Poverty Level (FPL)) could improve this far-reaching program in the aftermath of the current and during future emergencies that require similar restrictions.

To fill these gaps in the literature and inform the work of SNAP-Ed and similar programs, this study aimed to: (1) describe self-reported relative changes in food shopping and meal behaviors, dietary intake, and bodyweight from before to during the pandemic among low-income parents in California (with incomes at or below 185% of the FPL); (2) assess if these changes in food shopping and meal behaviors were associated with changes in dietary intake and bodyweight; and (3) assess the associations between the changes in dietary intake and changes in bodyweight.

## 2. Materials and Methods

### 2.1. Study Design and Sample Recruitment

This cross-sectional study examines data from an online survey of parents living within 185% of the FPL completed once between April and August of 2021. All applicable questions referenced the same time periods, including dates, when referring to “before the COVID-19 pandemic” (before March 2020) and “during the COVID-19 pandemic” (January–March 2021).

The study sample was recruited from 2363 parents who had completed a “media survey” between January and August 2021 conducted by the state health department to evaluate their SNAP-Ed social marketing campaign that included television/radio ads to promote healthy living. Survey recruitment was conducted through Facebook ads in English and Spanish in areas targeted by the social marketing campaign, including five California regions delineated by county (San Francisco Bay Area, Greater Sacramento, Northern California, San Joaquin Valley, and Southern California) and four zip code-delineated designated market areas (Fresno, Los Angeles, Sacramento, and San Diego) where residents receive similar local television and radio broadcasts [18,19]. Targeted survey recruitment and non-proportional quota sampling were used in an attempt to obtain approximately 15% African American, 50% Latino/a, and 30% White participants (there was no quota for people of other races/ethnicities) to approximate the racial/ethnic distribution of SNAP recipients in California.

Media survey recruitment ad respondents completed a self-administered Qualtrics online screener questionnaire between March 2019 and August 2021 (depending on when they were first recruited for media survey participation) that included questions regarding participant sociodemographics. Participants were eligible for media study inclusion if they were 18–59 years old, parent/guardian of at least one child 5–17 years old, had household income within 185% of the FPL, and preferred to speak English or Spanish.

Media survey participants who indicated a willingness to complete additional surveys received an email to participate in a self-administered online supplemental survey (a separate COVID-19-specific Qualtrics survey created for the current study) that took most respondents 20–25 min to complete. The supplemental survey included questions regarding respondent relative changes from prior to during the pandemic in food acquisition behaviors, dietary intake, and body weight (described in more detail in 2.2 Measures). Respondents received the participant bill of rights and online consent form describing the study purpose, survey question types, and that participation is voluntary and confidential. Supplemental survey participants received USD 40 if they had taken one media survey and USD 25 if they had participated in multiple media surveys.

Fraud detection software “Imperium” implemented within the survey using JavaScript code created a fraud profile and score for each respondent and screened out those who attempted to complete the survey multiple times. Those with a high fraud score were removed (n = 34).

Of the 2363 individuals invited to complete the supplemental survey, 1154 agreed to participate (48.8% response rate). After excluding 64 respondents with missing covariate or outcome data, the analytic sample included 1090 respondents.

### 2.2. Measures

Demographic data (used as covariates) obtained from the screener questionnaire included: race/ethnicity (included in the dataset if respondent chose African American, Asian/Pacific Islander, Latino/a, or White), gender (response options included male, female, or other, but only included in the dataset if respondent chose male or female), highest education level (high school graduate or less, some college, college graduate or more), adult age, and household size.

All other variables were derived from responses to supplemental survey questions and included relative change in frequency (from before to during the pandemic) of: (1) food shopping by venue (supermarket, small grocery, convenience store, discount store, farmer’s market, food bank/pantry) and method (in-store, ordered online for delivery, ordered online for pick-up); (2) meals by source (restaurant delivery, restaurant pick-up, charitable meals, meal kit delivery); (3) home meal practices (ate meals together as a household, time spent cooking, prepared healthy meals); (4) dietary intake (fruits and vegetables, SSBs, sweets, salty snacks, fast food, overall consumption); and (5) change in bodyweight.

With regard to change in food shopping frequency by venue type and method, respondents were asked about both frequency prior to (before March 2020) and during the pandemic (January–March 2021) for each of the venue types (supermarket, small grocery, convenience store, discount store, farmer’s market, food bank/pantry) and methods (in-store, ordered online for delivery, ordered online for pick-up). Response options for each question were never, sometimes, and often. Change in frequency of shopping at each venue type and for each method was determined by the difference in response to the questions regarding frequency before vs. during the pandemic and was coded as less than before the pandemic, about the same, and more than before the pandemic.

For questions regarding frequency of obtaining meals by source (restaurant delivery, restaurant pick-up, charitable meals, meal kit delivery), change in frequency of home meal practices (ate meals together as a household, time spent cooking, prepared healthy meals), change in frequency of intake of specific foods (fruits and vegetables, SSBs, sweets, salty snacks, fast food, overall consumption), and change in bodyweight, respondents were asked about each change in frequency from prior (before March 2020) to during the pandemic (January–March 2021) with response options less than before the pandemic, about the same, and more than before the pandemic and were coded as such.

Changes in food shopping, meal sources, and home meal practices served as independent variables, with changes in dietary intake and bodyweight as dependent variables (aim 2). Changes in dietary intake of select foods/beverages also served as independent variables, with overall consumption and bodyweight as dependent variables (aim 3).

Given the last-minute, unforeseen opportunity to survey this population during the pandemic, there was no opportunity to obtain pre-pandemic data, so we relied on participant recall of change over the time period of interest. To our knowledge, there are no validated questions regarding recall of relative change over time in food acquisition, bodyweight, and intake of specific foods, so we relied on questions developed by the research team for the purposes of this study. We used wording from other survey instruments [20,21,22,23] for items such as shopping venue types and shopping methods, household meal practices, and some aspects of question structure, but we had to restructure questions to address change over time and to reference the specific pandemic-related time period.

### 2.3. Statistical Analysis

Chi-square tests assessed demographic differences between those who completed the supplemental survey and those who declined or were excluded.

Descriptive statistics were used to describe self-reported relative changes in food shopping and meal behaviors, dietary intake, and bodyweight from before to during the pandemic.

Given the categorical nature of the dependent variables (that did not meet the proportional odds assumption), multinomial logistic regression models assessed associations among changes in food shopping and meal behaviors with the changes in dietary intake and bodyweight and changes in intake of specific types of food/beverages with change in overall food consumption and bodyweight. Each independent variable was modeled separately with each dependent variable. ‘About the same’ was used as the reference group for all change variables included in these models. All models were adjusted for age, race, gender, education, and household size. Bonferroni corrections for multiple comparisons were applied to the significance tests. Pseudo-R-squared values assessed model fit, which ranged from 0.03 to 0.348. Likelihood ratio chi-square tests assessed whether the overall models were statistically significant. All analyses were performed using RStudio Version 1.4.1717.

## 3. Results

### 3.1. Sample Description

Respondents were predominantly female (84.1%) and Latino/a (52.2%; Table 1). Forty-five percent of respondents had a high school diploma or less. Households had an average of five members. Those who declined participation in the supplemental survey or were excluded were statistically significantly less likely to be Latino/a, more likely to be White, came from smaller households, and were slightly older.

### 3.2. Relative Changes in Food Acquisition, Dietary Intake, and Bodyweight from before to during the Pandemic (Aim 1)

Many respondents reported that their intake of fruits and vegetables (54.1%), SSBs (44.4%), sweets (47.4%), salty snacks (51.8%), and fast food (28.9%; Table 2) had not changed. Fast food intake was the most likely to change, with 42.8% reporting a decrease and 28.3% reporting an increase. Similar proportions of respondents reported increases (26.5% and 24.9%, respectively) and decreases (26.0% and 23.2%, respectively) in sweets and salty snack intake. More respondents (27.7%) reported increases in fruit and vegetable intake than reported decreases (18.2%). More respondents reported increases in their overall food intake and bodyweight (34.6% and 46.2%, respectively) compared to decreases (20.1% and 16.1%, respectively).

Most respondents (61.9–68.6%) did not change their self-reported food shopping frequency by venue type. Decreases in shopping frequency were more common than increases at all shopping venue types except food banks/pantries (Table 2). Decreases in shopping frequency were most common at farmer’s markets (32.3% of respondents) and supermarkets (29.3%). Increases were most common at food banks/pantries (28.4%) and least common at farmer’s markets (5.8%).

While most respondents (57.5–63.2%, Table 2) did not report changing their frequency of food shopping by method, many increased their frequency of ordering food online, both for delivery (30.3%) and pick-up (27.2%). Decreased food shopping in-person (30.2%) was more prevalent than increased (6.6%).

Reported shifts away from restaurant use (decreased meal delivery and pick up, 42.7–43.3% of respondents respectively), and toward meals at home (increased meals eaten together (45.8%), household time spent cooking (51.0%), and household preparation of healthy meals (36.2%)) were common (Table 2). Very few respondents (6.2%) increased meal kit delivery.

### 3.3. Associations of Food Acquisition Behaviors with Changes in Dietary Intake and Bodyweight (Aim 2)

Compared to those with no change, those who decreased shopping at supermarkets and farmer’s markets had greater odds of decreased fruit and vegetable consumption (Odds Ratio (OR)[95%Confidence Interval (95%CI)] = 2.4[1.4–4.1] and OR[95%CI] = 1.8[1.0–3.1], respectively; Table 3). Those who decreased supermarket shopping also had greater odds of increased salty snack intake (OR[95%CI] = 1.7[1.0–2.8]). Those who decreased shopping at farmer’s markets also had greater odds of increased consumption of less healthy foods, including SSBs (OR[95%CI] = 1.9[1.1–3.2]) and sweets (OR[95%CI] = 1.8[1.1–2.9]) and increased bodyweight (OR[95%CI] = 1.7[1.1–2.6]). Those who increased shopping at food banks/pantries had greater odds of increased consumption of salty snacks (OR[95%CI] = [1.8]1.1–2.9).

Compared to those with no change, those who increased ordering food online for delivery had greater odds of increased intakes of sweets (OR[95%CI] = 1.7[1.1–2.8]), salty snacks (OR[95%CI] = 1.9[1.2–3.2]), fast food (OR[95%CI] = 2.0[1.2–3.5]) and food overall (OR[95%CI] = 1.9[1.2–3.0]), and increased bodyweight (OR[95%CI] = 1.8[1.1–2.9], Table 3). Similar associations were observed for ordering food online for pick up at a store. Those with decreased frequency of in-person food shopping had greater odds of increased intakes of sweets (OR[95%CI] = 1.9[1.2–3.1]) and salty snacks (OR[95%CI] = 1.9[1.2–3.1]), and increased bodyweight (OR[95%CI] = 2.1[1.3–3.4]).

Compared to those with no change, those who increased delivery of restaurant meals had greater odds of increased intakes of SSBs (OR[95%CI] = 2.3[1.2–4.4]), sweets, (OR[95%CI] = 2.7[1.5–4.8]) salty snacks (OR[95%CI] = 2.4[1.4–4.3], Table 3) and food overall (OR[95%CI] = 3.0[1.7–5.3]), and increased bodyweight (OR[95%CI] = 2.0[1.1–3.5]; Table 4). Similar associations were observed for increased restaurant meal pick-ups.

Compared to those with no change, those who increased eating meals together as a household had greater odds of reporting increased fruit and vegetable intake (OR[95%CI] = 2.0[1.3–3.2]), decreased fast food intake (OR[95%CI] = 2.0[1.3–3.5]), and increases in overall amount consumed (OR[95%CI] = 1.8[1.1–2.9]), and bodyweight (OR[95%CI] = 1.9[1.2–2.9]; Table 4). Those who increased household time spent cooking had greater odds of increased fruit and vegetable intake (OR[95%CI] = 2.3[1.4–3.7]) and decreased intakes of SSBs (OR[95%CI] = 2.4[1.5–3.8]), sweets (OR[95%CI] = 2.1[1.3–3.5]), salty snacks (OR[95%CI] = 1.8[1.1–3.1]), and fast food (OR[95%CI] = 2.6[1.6–4.2]) but results were mixed for overall food consumption and bodyweight. Those with increased household preparation of healthy meals had greater odds of increased fruit and vegetable intake (OR[95%CI] = 4.0[2.5–6.5] and decreased intakes of SSBs (OR[95%CI] = 3.7[2.3–6.0]), sweets (OR[95%CI] = 2.7[1.6–4.4]), salty snacks (OR[95%CI] = 3.0[1.8–5.1]), and fast food (OR[95%CI] = 2.8[1.7–4.6]), and decreased bodyweight (OR[95%CI] = 2.2[1.2–4.0]).

### 3.4. Associations of Changes in Dietary Intake with Changes in Bodyweight (Aim 3)

Compared to those with no change, those who reported increased fruit and vegetable consumption had greater odds of reporting decreased bodyweight (OR[95%CI] = 2.7[1.4–5.0], Table 5). Those who increased intakes of SSBs, sweets, and salty snacks had greater odds of increased overall food consumption (OR[95%CI] = 6.7[3.6–12.4]; OR[95%CI] = 8.9[5.0–15.7]; OR[95%CI] = 7.5[4.3–13.3], respectively). Similarly, those who increased intakes of SSBs, sweets, salty snacks, fast food, and more food overall had greater odds of increased bodyweight (OR[95%CI] = 3.5[1.9–6.3]; OR[95%CI] = 4.4[2.5–7.7]; OR[95%CI] = 3.7[2.1–6.3]; OR[95%CI] = 3.2[1.8–5.5]; OR[95%CI] = 10.1[5.8–17.5], respectively).

## 4. Discussion

This study examined relationships between self-reported changes from before to during the COVID-19 pandemic in food acquisition behaviors, dietary intake, and bodyweight among low-income parents in California, with the aim of informing nutrition policies and programs for low-income families and reducing health disparities in the aftermath of the current pandemic and during future emergencies that require similar restrictions.

The study findings suggest a promising trend toward improved dietary intake during the COVID-19 pandemic among the study population; more respondents reported increasing consumption of healthy foods and decreasing consumption of less healthy foods than vice versa. However, there was a more striking trend of increased overall food intake and bodyweight. A systematic review of changes in diet quality globally during the pandemic identified, by nation, heterogeneity and a slight improvement in overall diet quality—a result similar to this study’s findings [24]. The finding of increased bodyweight during the pandemic is aligned with national trends [25,26] and could be attributable to both diet-related factors [14]) and to demonstrated decreases in adult physical activity [27,28] during the pandemic. Although it is not possible to determine if the reported increases in overall food intake and weight were beneficial (i.e., within healthy weight for height ranges) or contributed to obesity, given the high prevalence of overweight and obesity in California [29,30,31], it is likely that much of the increases in overall food intake and weight could have contributed to overweight and obesity. Furthermore, the associations of the increases in weight and overall food intake with worsened dietary intakes suggest these changes were not healthy.

Respondents reported decreases in food shopping frequency at nearly all venues during the pandemic and at supermarkets and farmer’s markets, in particular, while also reporting increased online food shopping. These trends, which align with previous reports [14,31,32], could be due to concerns about COVID-19 exposure when shopping in-person and are substantiated by the accelerated growth of online food delivery businesses during COVID-19 [33], decreased farmer’s market sales [34], and decreased shopping in large supermarkets during the pandemic observed in a study of U.S. families with children [32]. The launching and expansion of a pilot program during the pandemic for online purchasing of foods with SNAP benefits may also have contributed to increases in online shopping among the low-income study population. The increase in food bank/pantry patronage could be explained by the large increases in unemployment due to COVID-19 restrictions that led to an unprecedented need for food assistance [35,36].

Our findings also suggest a shift away from restaurant meals and an increase in the preparation and consumption of meals at home. Similarly, a study in the U.S. found that only one-third of parents increased their online ordering from restaurants, and home cooking became much more common, during the COVID-19 pandemic [37]. Another study found that over 60% of U.S. adult respondents increased their home cooking frequency during the pandemic [16]. In addition to restaurant closures limiting on-site meals, studies have indicated that this shift from restaurant meals to home cooking may be due to a lack of confidence in restaurant food safety during the pandemic and the increased convenience of home cooking for parents spending more time at home [37,38]. Cost savings may also have been a motivation for decreased restaurant use, especially for those who experienced a job loss or reduction in hours.

Consistent with pre-pandemic studies, increases in online food ordering in this study were associated with worsened diet quality and increased bodyweight. A meta-analysis of U.S.-, Australia-, and Singapore-based studies found positive associations between online food delivery and the calorie, fat, and sodium content of the purchased foods [39]. Unhealthy food marketing on online food delivery sites could be a mechanism for these associations. A Brazilian study during the pandemic found that free online food delivery advertisements usually promoted unhealthy foods, such as pizza, candy, and salty snacks [40]. Studies suggest that addressing the promotion of healthier purchases via labeling, healthy default options, point-of-decision prompts, and product placement [39] during online food ordering could be effective at encouraging healthy choices. More research is needed on how marketing practices affect dietary choices when ordering food online and the cost-effectiveness of various intervention options.

The declines in the supermarket and farmer’s market shopping reported in the present study were also associated with worsened dietary intakes and increases in weight. While our study did not find associations between increased in-person shopping and improved diet quality, another study found that each additional visit to a grocery store during the pandemic was associated with improved Healthy Eating Index scores [41]. These findings suggest that, in addition to addressing the negative impacts of online food shopping, efforts to support in-person shopping by low-income parents at supermarkets and farmer’s markets may be warranted.

The finding that increased shopping at food banks/pantries was associated with an increase in salty snack consumption may be related to the quality of the available foods. Ensuring food banks/pantries are stocked with diverse and fresh foods is important for establishing equitable access to healthy foods [42], especially during and in the aftermath of public health emergencies.

Even though reported online ordering of restaurant meals decreased overall, increased online restaurant meal ordering, when it occurred, was associated with worsened dietary intakes and increases in weight. Conversely, decreases in restaurant meal ordering were associated with improvements in dietary intake. These trends are confirmed by pre-pandemic studies [43]. One study of U.S. adults found that the county-level availability of online food delivery through major platforms was associated with decreased cooking at home and increased BMI [44]. Since the online ordering of both meals and food had similar associations with changes in dietary intake and weight, these associations could be explained by the same mechanisms, including online food marketing. These concerns are echoed in a report on the potential dangers of online food and meal delivery platforms for public health, which identified an urgent need for studies on the impact of online food delivery [43].

While the reported increased frequencies of preparing and eating meals at home were associated with improved dietary intakes, they were also associated with weight gain. Only increased preparation of healthy foods at home was associated with both dietary improvements and decreases in weight. Pre-pandemic studies have identified positive associations between home cooking and diet quality [45,46]. Programs to support the continuation of these home meal practices may improve diets among low-income adults. However, similar to this study, other studies have found that home cooking not specifically focused on healthy foods was associated with weight gain [47]. A study during COVID-19 reported that increased home cooking was accompanied by increased saturated fat consumption and concluded that home cooking needs to focus on healthy food [17]. It is possible that increased economic insecurity during the pandemic made purchasing healthier options challenging [48]. Therefore, interventions to increase home cooking with healthy foods among low-income populations may be more effective if they include not only the provision of education and recipes but also increases in healthy food access (including price and quality) [49,50]. However, these suggested interventions should be considered in terms of the intervention cost (and possible unintended consequences) relative to the magnitude of the expected behavior change.

While this study explores change over time and compares those with different levels of naturally occurring change, this non-experimental design cannot establish causality. Furthermore, other factors such as changes in physical activity, employment status, food prices, and food availability that we did not account for could have contributed to the changes in dietary intake and/or bodyweight. However, unless these factors were causally related to both the exposure and outcomes in our models, they would not confound the observed associations. The study sample, although diverse, was not selected to be representative of all households with children in California living within 185% of FPL but rather focused on specific racial/ethnic groups living in areas that were past or future targets of media campaigns, thereby limiting the generalizability of the findings. The findings also cannot be generalized to other income, geographic, or racial/ethnic groups not included in this study. The media study participants were recruited through Facebook ads, which may introduce selection bias if those who respond to Facebook ads are different from those who do not. Due to the unexpected onset of COVID-19, this study relied on self-report of change over time, which may be subject to recall error and/or social desirability bias [51]. By using relative change rather than absolute values pre and during the pandemic, we are not able to determine the adequacy of the respondents’ diets or bodyweight at either time point. Self-reports of bodyweight and dietary intake are subject to various biases and errors, some of which are directional and vary depending on the type of food recalled, the recall methodology employed, and respondent characteristics (i.e., gender, age, and weight status) [52,53,54]. However, in this study, the sudden and dramatic pandemic-driven changes served to clearly demarcate the comparison time periods, thereby facilitating respondent recall of change. Furthermore, the categorical response options did not require a high degree of recall precision, and many of the findings are consistent with other studies.

## 5. Conclusions

This study found that among low-income California parents, increases in online food and meal shopping during COVID-19 were associated with worsened dietary intakes and increased bodyweight, while increases in healthy home meal preparation and decreases in restaurant meals were associated with improved dietary intakes and weight loss. Given the persistence of COVID-19 and related economic and food environment changes, it is plausible that these trends in food acquisition will persist, at least in part. To protect health and avoid exacerbating health inequities, nutrition programs and policies to address the potential negative influence of ordering food and meals online and the potential positive influence of healthy home-cooking practices on diet and weight may be needed in the aftermath of the current pandemic. These findings may also help inform preparation for, and responses to, future pandemics and emergencies, such as climate-related fires, heat events, and floods that may require similar restrictions and are likely to become more common.

## Figures and Tables

**Table 1:** Characteristics of sampled low-income ^a^ Californian parents (n = 1090) compared to those who declined survey participation or were excluded (n = 1273).

	Analytic Sample (n = 1090)	Declined Participation or Were Excluded (n = 1273) ^b^	Chi-Squared *p*-Value
	n (%)		
Gender			0.637
Female	917 (84.1)	1081 (84.9)	
Male	173 (15.9)	192 (15.1)	
Race/Ethnicity			<0.001
Latino/a	569 (52.2)	582 (45.7)	
White/Caucasian	333 (30.6)	487 (38.3)	
African American	114 (10.5)	144 (11.3)	
Asian/Pacific Islander	74 (6.8)	60 (4.7)	
Highest education level			0.003
High school graduate/GED or less	492 (45.1)	585 (46.4)	
Some college or Associate degree	410 (37.6)	519 (41.2)	
College graduate or higher	188 (17.2)	156 (12.4)	
	Mean (standard deviation)		
Age	37.8 (7.9)	39.4 (8.1)	<0.001
Household size	5.0 (1.8)	4.8 (1.8)	0.01
Number of children in the household	2.6 (1.3)	2.3 (1.2)	<0.001

^a^ Income at or below 185% of the Federal Poverty Level; ^b^ 1209 declined participation; 64 were excluded due to missing covariate or outcome data.

**Table 2:** Self-reported relative changes from before to during the COVID-19 pandemic in dietary intake, bodyweight, and food acquisition among the sample of low-income ^a^ parents in California (n = 1090).

	Relative Change from Pre-Pandemic (Prior to March 2020) to during the Pandemic (January–March 2021)		
Behaviors	Decreased	Stayed about the Same	Increased
	n (%)	n (%)	n (%)
Dietary intake frequency			
Fruit and vegetable	198 (18.2)	590 (54.1)	302 (27.7)
Sugar-sweetened beverages	385 (35.3)	484 (44.4)	221 (20.3)
Sweets	284 (26.1)	517 (47.4)	289 (26.5)
Salty snack	253 (23.2)	565 (51.8)	272 (24.9)
Fast food	466 (42.8)	315 (28.9)	309 (28.3)
Overall	219 (20.1)	494 (45.3)	377 (34.6)
Bodyweight	176 (16.1)	410 (37.6)	504 (46.2)
Food shopping frequency by venue			
Supermarket	309 (29.3)	696 (63.9)	85 (7.8)
Small grocery	236 (21.7)	721 (66.1)	133 (12.2)
Convenience store	223 (20.5)	742 (68.1)	125 (11.5)
Discount store	245 (22.5)	748 (68.6)	97 (8.9)
Farmer’s market	352(32.3)	675 (61.9)	63 (5.8)
Food bank/pantry	77(7.1)	703 (64.5)	310 (28.4)
Food shopping frequency by method			
Ordering online, delivered to home	133 (12.2)	627 (57.5)	330 (30.3)
Ordering online, picked up at store	143 (13.1)	650 (59.6)	297 (27.2)
In-person	329 (30.2)	689 (63.2)	72 (6.6)
Meal frequency by source			
Restaurant delivery	465 (42.7)	380 (34.9)	245 (22.5)
Restaurant pick up	472 (43.3)	355 (32.6)	263 (24.1)
Charitable meals	489 (44.9)	446 (40.9)	155 (14.2)
Meal kit delivered to home	575 (52.8)	447 (41.0)	68 (6.2)
Frequency of home meal practices			
Ate meals together as a household	122 (11.2)	469 (43.0)	499 (45.8)
Spent time cooking	135 (12.4)	399 (36.6)	556 (51.0)
Prepared healthy meals	171 (15.7)	524 (48.1)	395 (36.2)

^a^ Income at or below 185% of the Federal Poverty Level.

**Table 3:** Adjusted multinomial associations of self-reported relative change in food shopping behaviors with self-reported change in dietary intake and bodyweight from before to during the COVID-19 pandemic ^a^ among the sample of low-income parents ^b^ in California (n = 1090) ^c,d^.

		Relative Change													
		Fruit and Vegetable Consumption OR(95%CI)		Sugar-Sweetened Beverage Consumption OR(95%CI)		Sweets Consumption OR(95%CI)		Salty Snack Consumption OR(95%CI)		Fast Food Consumption OR(95%CI)		Overall Consumption OR(95%CI)		Bodyweight OR (95%CI)	
		Decreased	Increased	Decreased	Increased	Decreased	Increased	Decreased	Increased	Decreased	Increased	Decreased	Increased	Decreased	Increased
**Relative change in food shopping frequency by venue type** (ref. “About the same as before the pandemic”)															
Supermarket	Decreased	**2.4**	1.1	1.1	1.7	1.5	1.4	1.5	**1.7**	1.3	1.3	1.4	1	1.8	1.4
		**(1.4, 4.1)**	(0.6, 1.8)	(0.6, 1.7)	(<1.0, 2.9)	(0.9, 2.5)	(0.8, 2.3)	(0.8, 2.5)	**(1.0, 2.8)**	(0.7, 2.1)	(0.7, 2.3)	(0.8, 2.5)	(0.6, 1.7)	(0.9, 3.3)	(0.9, 2.3)
	Increased	1.5	1.5	1.2	1.4	1.6	0.9	0.9	0.9	0.6	0.8	1.4	0.7	2.5	1.3
		(0.6, 4.2)	(0.7, 3.4)	(0.5, 2.8)	(0.5, 3.5)	(0.7, 3.6)	(0.3, 2.2)	(0.4, 2.4)	(0.3, 2.1)	(0.2, 1.4)	(0.3, 2.0)	(0.6, 3.4)	(0.3, 1.6)	(0.9, 6.6)	(0.6, 2.9)
Small grocery	Decreased	1.5	1	0.8	1.4	1.3	1.1	1.1	1.1	1	1.3	**1.9**	1.2	1.3	1
		(0.8, 2.7)	(0.6, 1.8)	(0.5, 1.4)	(0.8, 2.5)	(0.7, 2.2)	(0.6, 2.0)	(0.6, 1.9)	(0.6, 1.9)	(0.6, 1.8)	(0.7, 2.4)	**(1.0, 3.4)**	(0.7, 2.1)	(0.7, 2.6)	(0.6, 1.6)
	Increased	1.3	1	1.2	1.3	1.1	0.9	0.8	1.2	1.2	1.7	1.1	1.2	1.6	1.8
		(0.6, 2.8)	(0.5, 1.9)	(0.6, 2.3)	(0.6, 2.9)	(0.5, 2.1)	(0.4, 1.9)	(0.4, 1.8)	(0.6, 2.4)	(0.6, 2.4)	(0.8, 3.7)	(0.5, 2.4)	(0.6, 2.3)	(0.6, 3.9)	(0.9, 3.6)
Convenience store	Decreased	1.1	1.3	1.2	0.9	1.2	0.8	1.1	0.9	1	0.9	1.3	0.9	1.6	1.2
		(0.6, 2.1)	(0.8, 2.2)	(0.7, 2.0)	(0.5 1.8)	(0.7, 2.1)	(0.5, 1.5)	(0.6, 2.0)	(0.5, 1.7)	(0.6, 1.8)	(0.5, 1.7)	(0.7, 2.4)	(0.5, 1.6)	(0.8, 3.2)	(0.7, 2.0)
	Increased	2	1	0.9	1.3	1.1	1.2	1.3	1.5	1.1	1.3	1.3	1	1.2	1.6
		(<1.0,4.2)	(0.5, 2.1)	(0.5, 1.9)	(0.6, 2.7)	(0.5, 2.3)	(0.6, 2.4)	(0.6, 2.8)	(0.7, 3.1)	(0.5, 2.4)	(0.6, 2.9)	(0.6, 2.8)	(0.5, 1.9)	(0.5, 3.1)	(0.8, 3.2)
Discount store	Decreased	1.4	0.8	1	1.6	1.4	1.5	1	1.4	1.4	1.4	1.3	1.5	1.1	1.5
		(0.8, 2.6)	(0.5, 1.4)	(0.6, 1.7)	(0.9, 2.8)	(0.8, 2.5)	(0.9, 2.7)	(0.5, 1.8)	(0.8, 2.3)	(0.8, 2.4)	(0.8, 2.6)	(0.7, 2.4)	(0.9, 2.4)	(0.6, 2.3)	(0.9, 2.4)
	Increased	1.8	1.2	1.5	1.2	1.2	1	0.9	0.8	1	0.9	1.5	1.1	1.4	1.1
		(0.8, 4.3)	(0.6, 2.7)	(0.7, 3.3)	(0.5, 3.1)	(0.5, 2.7)	(0.4, 2.3)	(0.4, 2.0)	(0.3, 1.8)	(0.5, 2.2)	(0.3, 2.1)	(0.7, 3.5)	(0.5, 2.4)	(0.5, 3.5)	(0.5, 2.4)
Farmer’s market	Decreased	**1.8**	1.4	1.2	**1.9**	1.1	**1.8**	0.7	1.4	1.2	1.5	1.4	1.5	1.2	**1.7**
		**(1.0, 3.1)**	(0.9, 2.2)	(0.8, 2.0)	**(1.1, 3.2)**	(0.7, 1.9)	**(1.1, 2.9)**	(0.4, 1.3)	(0.9, 2.3)	(0.7, 2.0)	(0.9, 2.7)	(0.8, 2.4)	(<1.0, 2.4)	(0.6, 2.3)	**(1.1, 2.6)**
	Increased	0.9	1.4	1.2	0.7	1.4	0.6	1.5	1	0.9	1	1	1	1.7	1.2
		(0.2, 3.1)	(0.5, 3.3)	(0.5, 3.0)	(0.2, 2.4)	(0.6, 3.4)	(0.2, 1.9)	(0.6, 3.8)	(0.3, 2.8)	(0.3, 2.3)	(0.3, 2.8)	(0.3, 3.1)	(0.4, 2.5)	(0.6, 5.2)	(0.5, 2.9)
Food bank/pantry	Decreased	1.1	1.1	1	1.2	1.2	1.2	1.6	1.5	1.4	1.4	1.9	1.2	1	0.8
		(0.4, 3.0)	(0.4, 2.5)	(0.4, 2.3)	(0.4, 3.0)	(0.5, 2.9)	(0.5, 2.9)	(0.6, 4.0)	(0.6, 3.8)	(0.6, 3.6)	(0.5, 3.7)	(0.7, 5.0)	(0.5, 3.0)	(0.3, 2.9)	(0.4, 1.9)
	Increased	1.6	1.5	1.2	1.7	1.3	1.4	1.2	**1.8**	1.5	1.3	1.7	1.6	1.4	1.5
		(0.9, 2.7)	(0.9, 2.4)	(0.7, 2.0)	(<1.0, 3.0)	(0.8, 2.1)	(0.9, 2.4)	(0.7, 2.1)	**(1.1, 2.9)**	(0.9, 2.5)	(0.7, 2.2)	(<1.0, 2.9)	(<1.0, 2.6)	(0.7, 2.6)	(0.9, 2.3)
**Relative change in food shopping frequency by method** (ref. “About the same as before the pandemic”)															
Ordered online, delivered to home	Decreased	1.1	1.3	1	1.6	1.3	1	1.4	1.1	1.4	1.5	1.1	1	1.4	1.5
		(0.5, 2.4)	(0.6, 2.5)	(0.5, 2.0)	(0.7, 3.5)	(0.6, 2.4)	(0.4, 2.2)	(0.7, 2.8)	(0.5, 2.6)	(0.7, 2.9)	(0.6, 3.5)	(0.5, 2.2)	(0.5, 2.0)	(0.6, 3.4)	(0.7, 2.8)
	Increased	1.1	0.8	0.9	1.6	0.9	**1.7**	0.7	**1.9**	0.9	**2**	0.9	**1.9**	1.1	**1.8**
		(0.6, 1.9)	(0.5, 1.4)	(0.5, 1.4)	(0.9, 2.8)	(0.5, 1.6)	**(1.1, 2.8)**	(0.4, 1.3)	**(1.2, 3.2)**	(0.5, 1.5)	**(1.2, 3.5)**	(0.5,1.7)	**(1.2, 3.0)**	(0.5, 2.0)	**(1.1, 2.9)**
Ordered online, picked up at store	Decreased	1	1.3	1	1.1	1.4	0.8	1.2	0.8	0.9	0.7	1.1	1.1	1.7	1.2
		(0.4, 2.2)	(0.7, 2.5)	(0.5, 1.9)	(0.5, 2.4)	(0.7, 2.6)	(0.4, 1.7)	(0.6, 2.3)	(0.3, 1.7)	(0.5, 1.8)	(0.3, 1.6)	(0.5, 2.2)	(0.5, 2.1)	(0.7, 3.7)	(0.6, 2.2)
	Increased	1.2	1.1	1	1.7	1.1	**1.7**	0.9	**1.8**	0.9	1.6	1	**1.8**	1.4	**2.1**
		(0.7, 2.2)	(0.7, 1.9)	(0.6, 1.6)	(<1.0, 3.0)	(0.6, 2.0)	**(1.0, 2.8)**	(0.5, 1.6)	**(1.1, 3.0)**	(0.5, 1.5)	(0.9, 2.8)	(0.5,1.9)	**(1.1, 3.0)**	(0.7, 2.8)	**(1.2, 3.4)**
In-person	Decreased	1.7	1	0.8	1.7	1.2	**1.9**	1.1	**1.9**	1	1.3	0.9	1.2	1.9	**2.1**
		(<1.0, 2.9)	(0.6, 1.6)	(0.5, 1.3)	(1.0, 2.9)	(0.7, 2.0)	**(1.2, 3.1)**	(0.7, 2.0)	**(1.2, 3.1)**	(0.6, 1.6)	(0.8, 2.3)	(0.5, 1.5)	(0.8, 2.0)	(<1.0, 3.5)	**(1.3, 3.4)**
	Increased	0.8	1	0.8	1.5	1	1.1	0.8	0.9	0.6	1	0.8	1.1	1.4	1.5
		(0.2, 2.6)	(0.4, 2.3)	(0.3, 1.9)	(0.6, 4.1)	(0.4, 2.5)	(0.4, 2.8)	(0.3, 2.1)	(0.3, 2.3)	(0.2, 1.6)	(0.4, 2.7)	(0.3, 2.3)	(0.5, 2.6)	(0.4, 4.3)	(0.6, 3.4)

OR, odds ratio; CI, confidence interval. Bold font indicates results significant at *p* < 0.05. ^a^ From prior to March 2020 to January–March 2021. ^b^ Income at or below 185% of the Federal Poverty Level. ^c^ All models adjusted for age, race, gender, household size, and education and include Bonferroni *p*-value correction. ^d^ Likelihood ratio chi-square test *p*-values were less than 0.05 for all models in this table.

**Table 4:** Adjusted multinomial associations of relative change in self-reported meal behaviors with self-reported changes in dietary intake and bodyweight from before to during the COVID-19 pandemic ^a^ among the sample of low-income ^b^ parents in California (n = 1090) ^c,d^.

Relative Change															
		Fruit and Vegetable Consumption, OR(95%CI)		Sugar-Sweetened Beverage Consumption, OR(95%CI)		Sweets Consumption, OR(95%CI)		Salty Snack Consumption, OR(95%CI)		Fast Food Consumption OR(95%CI)		Overall Consumption OR(95%CI)		Bodyweight OR(95%CI)	
		Decreased	Increased	Decreased	Increased	Decreased	Increased	Decreased	Increased	Decreased	Increased	Decreased	Increased	Decreased	Increased
**Relative change in meal frequency by source** (ref. “About the same as before the pandemic”)															
Restaurant delivery	Decreased	**2.2 (1.1,4.1)**	1.4	**2.3**	1.6	**2.8**	1.5	**3**	1.2	**4.4**	**2.4**	**3.1**	1.4	1.9	1.2
			(0.8, 2.3)	**(1.4, 3.8)**	(0.9, 3.0)	**(1.6, 4.8)**	(0.8, 2.6)	**(1.7, 5.3)**	(0.7, 2.1)	**(2.6, 7.6)**	**(1.3, 4.4)**	**(1.8, 5.7)**	(0.8, 2.3)	(<1.0, 3.6)	(0.8, 2.0)
	Increased	**2.7**	1.6	1.1	**2.3**	1.3	**2.7**	1.3	**2.4**	1.8	**4.7**	2	**3**	1.4	**2**
		**(1.3, 5.5)**	(0.9, 3.0)	(0.6, 2.1)	**(1.2, 4.4)**	(0.6, 2.6)	**(1.5, 4.8)**	(0.6, 2.8)	**(1.4, 4.3)**	(0.9, 3.5)	**(2.5, 9.1)**	(0.9, 4.5)	**(1.7, 5.3)**	(0.6, 3.3)	**(1.1, 3.5)**
Restaurant pick-up	Decreased	**2**	1.7	**2.4**	1.5	**2.7**	1.2	**3.8**	0.9	**3.9**	1.5	**2.7**	1.1	2	1.1
		**(1.0, 3.8)**	(<1.0, 2.8)	**(1.5, 4.0)**	(0.8, 2.8)	**(1.6, 4.7)**	(0.7, 2.0)	**(2.1, 7.1)**	(0.5, 1.5)	**(2.3, 6.6)**	(0.8, 2.8)	**(1.5, 5.1)**	(0.6, 1.8)	(<1.0, 3.8)	(0.7, 1.8)
	Increased	**2.7**	1.5	1.1	**2.3**	1.3	**2.7**	1.6	**2.3**	1.4	**4**	1.9	**2.9**	2.1	**2.3**
		**(1.4, 5.5)**	(0.8, 2.7)	(0.6, 2.1)	**(1.2, 4.3)**	(0.6, 2.6)	**(1.5, 4.9)**	(0.7, 3.5)	**(1.3, 4.0)**	(0.7, 2.7)	**(2.1, 7.5)**	(0.8, 4.2)	**(1.6, 5.0)**	(0.9, 4.7)	**(1.3, 4.0)**
Charitable meals	Decreased	1.6	1.4	1.5	1.2	**1.7**	1.4	**1.7**	1.1	**1.7**	**1.9**	1.3	1.3	1.2	1.1
		(0.9, 2.7)	(0.8, 2.2)	(0.9, 2.4)	(0.7, 2.1)	**(1.0, 2.9)**	(0.8, 2.3)	**(1.0, 3.0)**	(0.7, 1.9)	**(1.1, 2.9)**	**(1.1, 3.2)**	(0.8, 2.3)	(0.8, 2.0)	(0.7, 2.3)	(0.7, 1.8)
	Increased	1.8	1.7	1.5	1.8	1.2	1.6	**2.4**	**2.1**	1.9	2	1.7	1.7	1.8	1.7
		(0.8, 3.8)	(0.9, 3.2)	(0.8, 3.0)	(0.9, 3.9)	(0.6, 2.6)	(0.8, 3.2)	**(1.1, 5.1)**	**(1.1, 4.2)**	(0.9, 3.9)	(0.9, 4.4)	(0.8, 3.6)	(0.9, 3.3)	(0.8, 4.3)	(0.9, 3.3)
Meals kits delivered to home	Decreased	1.3	1.4	**1.8**	1.2	1.4	1.2	**1.9**	1	**2.1**	**1.8**	1.4	1.2	1.3	1
		(0.7, 2.2)	(0.9, 2.2)	**(1.1, 2.9)**	(0.7, 2.0)	(0.8, 2.2)	(0.7, 2.0)	**(1.1, 3.3)**	(0.6, 1.7)	**(1.3, 3.5)**	**(1.0, 3.0)**	(0.8, 2.4)	(0.7, 1.9)	(0.7, 2.3)	(0.7, 1.6)
	Increased	1.2	1.7	1.7	0.9	1.2	1.6	1.5	1.6	1.2	1.2	2.4	1.9	1.9	1.5
		(0.4, 3.8)	(0.7, 4.2)	(0.7, 4.2)	(0.3, 2.9)	(0.4, 3.4)	(0.6, 4.1)	(0.5, 4.5)	(0.6, 4.1)	(0.5, 3.2)	(0.4, 3.5)	(0.8, 6.9)	(0.7, 4.8)	(0.6, 6.1)	(0.6, 3.8)
**Relative change in frequency of home meal practices** (ref. “About the same as before the pandemic”)															
Ate meals together as a household	Decreased	**4.3**	1.5	1.3	2	1.4	1.3	1.4	1	1.9	**2.5**	2.2	2.2	1.7	1.9
		**(2.0, 9.1)**	(0.7, 3.4)	(0.6, 2.8)	(0.9, 4.4)	(0.7, 3.1)	(0.6, 2.9)	(0.6, 3.0)	(0.5, 2.3)	(0.8, 4.4)	**(1.1, 5.8)**	(0.9, 5.0)	(1.0, 4.5)	(0.7, 4.2)	(0.9, 3.8)
	Increased	1.5	**2**	**2.1**	**1.8**	**2**	**1.8**	1.6	1.6	**2.1**	1.5	1.4	**1.8**	1.5	**1.9**
		(0.8, 2.6)	**(1.3, 3.2)**	**(1.3, 3.3)**	**(1.0, 3.0)**	**(1.2, 3.3)**	**(1.1, 3.0)**	(0.9, 2.6)	(<1.0, 2.6)	**(1.3, 3.5)**	(0.9, 2.6)	(0.8, 2.4)	**(1.1, 2.9)**	(0.8, 2.7)	**(1.2, 2.9)**
Spent time cooking	Decreased	**5**	1.4	1.7	**3.6**	2	**2.9**	1.6	**2.7**	1.6	**4.8**	**3.1**	**4.6**	1.6	**2.3**
		**(2.3, 10.5)**	(0.6, 3.3)	(0.8, 3.8)	**(1.7, 7.8)**	(0.9, 4.6)	**(1.4, 5.9)**	(0.7, 3.8)	**(1.3, 5.5)**	(0.6, 4.0)	**(2.1, 10.9)**	**(1.3, 7.7)**	**(2.2, 9.9)**	(0.6, 4.1)	**(1.1, 4.5)**
	Increased	1.5	**2.3**	**2.4**	1.5	**2.1**	1.6	**1.8**	1.6	**2.6**	1.2	1.5	1.6	1.5	**1.6**
		(0.8, 2.7)	**(1.4, 3.7)**	**(1.5, 3.8)**	(0.9, 2.7)	**(1.3, 3.5)**	(0.9, 2.6)	**(1.1, 3.1)**	(0.9, 2.7)	**(1.6, 4.2)**	(0.7, 2.1)	(0.8, 2.5)	(<1.0, 2.5)	(0.8, 2.6)	**(1.0, 2.5)**
Prepared healthy meals	Decreased	**6.8**	1.5	1.6	**4.5**	1.7	**3.1**	1.9	**3.5**	2.1	**5.9**	**2.8**	**4.5**	2	**3.3**
		**(3.5, 13.2)**	(0.6, 3.4)	(0.8, 3.3)	**(2.3, 8.8)**	(0.8, 3.6)	**(1.6, 5.8)**	(0.9, 4.3)	**(1.9, 6.6)**	(0.9, 4.8)	**(2.7, 13.0)**	**(1.2, 6.5)**	**(2.3, 8.8)**	(0.8, 5.4)	**(1.7, 6.4)**
	Increased	0.9	**4**	**3.7**	1.5	**2.7**	1.2	**3**	1.3	**2.8**	1	1.7	1	**2.2**	1.2
		(0.5, 1.9)	**(2.5, 6.5**)	**(2.3, 6.0)**	(0.8, 2.9)	**(1.6, 4.4)**	(0.7, 1.9)	**(1.8, 5.1)**	(0.7, 2.2)	**(1.7, 4.6)**	(0.5, 1.8)	(1.0, 3.0)	(0.6, 1.6)	**(1.2, 4.0)**	(0.8, 1.9)

OR, odds ratio; CI, confidence interval. Bold font indicates results significant at *p* < 0.05. ^a^ Comparing the time periods before March 2020 to January–March 2021. ^b^ Income at or below 185% of the Federal Poverty Level. ^c^ All models adjusted for age, race, gender, household size, and adult education and include Bonferroni *p*-value correction. ^d^ Likelihood ratio chi-square test *p*-values were less than 0.05 for all models in this table.

**Table 5:** Adjusted multinomial associations of self-reported relative changes in dietary intake with self-reported relative change in overall amount of food consumed and bodyweight from before to during the COVID-19 pandemic ^a^ among the sample of low-income ^b^ parents in California (n = 1090) ^c,d^.

		Relative Change			
		Overall Consumption OR (95%CI)		Bodyweight OR (95%CI)	
		Decreased	Increased	Decreased	Increased
**Relative change in dietary intake** (ref. “About the same as before the pandemic”)					
Fruit and vegetable	Decreased	**2.8 (1.4, 5.7)**	**2.9 (1.6, 5.3)**	1.7 (0.7, 4.0)	**2.4 (1.3, 4.3)**
	Increased	**2.1 (1.2, 3.8)**	**1.7 (1.0, 2.8)**	**2.7 (1.4, 5.0)**	1.4 (0.9, 2.3)
Sugar-sweetened beverages	Decreased	**2.9 (1.7, 5.1)**	1.0 (0.6, 1.6)	**3.0 (1.6, 5.7)**	0.8 (0.5, 1.3)
	Increased	2.2 (0.9, 5.3)	**6.7 (3.6, 12.4)**	1.3 (0.5, 3.8)	**3.5 (1.9, 6.3)**
Sweets	Decreased	**3.4 (2.0, 6.0)**	0.8 (0.4, 1.6)	**2.5 (1.3, 4.6)**	0.9 (0.5, 1.5)
	Increased	1.5 (0.6, 3.5)	**8.9 (5.0, 15.7)**	1.2 (0.5, 2.9)	**4.4 (2.5, 7.7)**
Salty snacks	Decreased	**2.7 (1.5, 4.8)**	0.6 (0.3, 1.2)	**2.7 (1.4, 5.2)**	0.7 (0.4, 1.2)
	Increased	1.6 (0.7, 3.7)	**7.5 (4.3, 13.3)**	1.4 (0.6, 3.2)	**3.7 (2.1, 6.3)**
Fast food	Decreased	**5.4 (2.7, 10.8)**	1.4 (0.8, 2.5)	**2.2 (1.1, 4.2)**	1.5 (0.9, 2.4)
	Increased	**2.5 (1.0, 6.3)**	**6.9 (3.8, 12.4)**	1.1 (0.4, 2.5)	**3.2 (1.8, 5.5)**
Overall	Decreased	-	-	**7.2 (3.6, 14.1)**	1.4 (0.7, 2.7)
	Increased	-	-	1.6 (0.6, 4.0)	**10.1 (5.8, 17.5)**

OR, odds ratio; CI, confidence interval. Bold font indicates results significant at *p* < 0.05. ^a^ From prior to March 2020 to January–March 2021. ^b^ Income at or below 185% of the Federal Poverty Level. ^c^ All models adjusted for age, race, gender, household size, and education and include Bonferroni value correction. ^d^ Likelihood ratio chi-square test *p*-values were less than 0.001 for all models in this table.

## Data Availability

The data presented in this study are available on request from the corresponding author. The data are not publicly available to protect privacy and comply with approved protocols.
